# Modeling Long-Term Postseismic Deformation Following the 2020 Mw 7.0 Samos Earthquake Using Campaign and Continuous GNSS Observations

**DOI:** 10.3390/s26175609

**Published:** 2026-09-03

**Authors:** Halil İbrahim Solak, İbrahim Tiryakioğlu, Cemil Gezgin, Kayhan Aladoğan, Sefa Yalvaç, Bahadır Aktuğ, Cemal Özer Yiğit, Ergin Dönmez, Ertuğrul Demirelli, Eda Esma Eyübagil, Ece Bengünaz Çakanşimşek Ünlükaya, Furkan Şahiner, Muhiddin Can Yıldırım, Vahap Engin Gülal

**Affiliations:** 1 Vocational School of Information Technologies, Afyon Kocatepe University, 03200 Afyonkarahisar, Türkiye; hisolak@aku.edu.tr; 2 Earthquake Implementation and Research Center, Afyon Kocatepe University, 03200 Afyonkarahisar, Türkiye; itiryakioglu@aku.edu.tr; 3 Department of Geomatics Engineering, Afyon Kocatepe University, 03200 Afyonkarahisar, Türkiye; 4 Department of Geomatics Engineering, Aksaray University, 68100 Aksaray, Türkiye; cemilgezgin@aksaray.edu.tr; 5 Osmancık Ömer Derindere Vocational School, Hitit University, 19030 Çorum, Türkiye; kayhanaladogan@hitit.edu.tr; 6 Department of Geomatics, Gumushane University, 29830 Gumushane, Türkiye; sefayalvac@gumushane.edu.tr; 7 Department of Geophysical Engineering, Ankara University, 06830 Ankara, Türkiye; aktug@ankara.edu.tr; 8 MDSoft Informatics, Ankara University Technopolis, 06830 Ankara, Türkiye; 9 Department of Geomatics Engineering, Gebze Technical University, 41400 Gebze, Türkiye; cyigit@gtu.edu.tr; 10 Çivril Atasay Kamer Vocational School, Pamukkale University, 20160 Denizli, Türkiye; ergindonmez@gmail.com; 11 Graduate School of Natural and Applied Sciences, Afyon Kocatepe University, 03200 Afyonkarahisar, Türkiye; ertugruldemirelli@gmail.com (E.D.); edaeyubagil@gmail.com (E.E.E.); ece-bengunaz.cakansimsek@usr.aku.edu.tr (E.B.Ç.Ü.); frnsah44@gmail.com (F.Ş.); yildirimmuhiddincan@gmail.com (M.C.Y.); 12 Department of Computer Engineering, Atlas University, 34408 İstanbul, Türkiye

**Keywords:** postseismic deformation, Samos earthquake, campaign GNSS, time-series modeling, Western Anatolia

## Abstract

**Highlights:**

**What are the main findings?**

- Long-term postseismic deformation following the 2020 Mw 7.0 Samos earthquake was successfully characterized using integrated campaign and continuous GNSS observations spanning approximately 4.5 years.
- An AICc-based model-selection framework combined with Monte Carlo observation–perturbation analysis identified 18 linear, 9 logarithmic, 7 exponential, and 2 combined logarithmic–exponential solutions. The nonlinear solutions passing the Monte Carlo and goodness-of-fit criteria showed single-process characteristic times of approximately 183–731 days, while the LOGEXP solutions additionally contained a shorter logarithmic timescale of approximately 110 days.

**What are the implications of the main findings?**

- The results demonstrate that campaign GNSS observations, when supported by robust statistical model selection, can provide meaningful constraints on long-term postseismic deformation despite sparse temporal sampling.
- The observed spatial variability highlights that postseismic deformation is strongly time-dependent and tectonically heterogeneous, emphasizing the importance of appropriate observation timing for improving earthquake-cycle studies and geodetic hazard assessments.

**Abstract:**

Postseismic deformation provides fundamental insights into earthquake-cycle processes, lithospheric rheology, and stress redistribution following large earthquakes. Although the 2020 Mw 7.0 Samos earthquake has been extensively investigated in terms of coseismic deformation and early postseismic behavior, the long-term evolution of deformation following the event remains poorly constrained. This study characterizes the long-term spatiotemporal evolution of postseismic deformation associated with the 2020 Mw 7.0 Samos earthquake using combined campaign and continuous GNSS observations. A total of 18 GNSS stations were analyzed over an approximately 4.5-year period following the earthquake. Pre-earthquake GNSS velocities were incorporated as prior constraints, while postseismic deformation was modeled using linear, logarithmic, exponential, and combined logarithmic–exponential functions. The preferred model for each station component was identified using the corrected Akaike Information Criterion (AICc), and model-selection robustness was evaluated through 1000 Monte Carlo observation–perturbation simulations. The results reveal a spatially heterogeneous postseismic deformation field with station-dependent temporal behavior. Among the nonlinear solutions passing the Monte Carlo and goodness-of-fit criteria, the seven single-process LOG and EXP solutions yielded characteristic relaxation times ranging from 182.6 to 730.5 days, with a median of 438.3 days. The two LOGEXP solutions additionally contained a logarithmic timescale of 109.6 days and exponential timescales of 292.2–438.3 days. These findings demonstrate that campaign GNSS observations, when integrated with continuous GNSS data and an objective statistical framework, can provide meaningful constraints on the long-term evolution of postseismic deformation despite sparse temporal sampling. More broadly, the results emphasize the strongly time-dependent nature of postseismic deformation and the critical role of observation timing in capturing its spatiotemporal evolution.

## 1. Introduction

Earthquakes are driven by the abrupt release of elastic strain accumulated along active faults, generating crustal deformation over a broad range of temporal scales. This deformation extends well beyond coseismic fault rupture and encompasses the successive stages of the earthquake cycle, including interseismic strain accumulation, coseismic displacement, and postseismic deformation. During the interseismic phase, tectonic loading progressively accumulates shear stress along locked fault segments until it is suddenly released during an earthquake, generating seismic waves that propagate through the surrounding crust [1]. Following the mainshock, the crust undergoes a prolonged period of deformation and mechanical readjustment as it responds to the perturbed stress field. Surface displacements observed during this postseismic adjustment reflect the combined effects of multiple physical mechanisms, including afterslip along the fault plane, viscoelastic relaxation within the lower crust and upper mantle, and poroelastic rebound. Afterslip represents the continued aseismic slip on the coseismic rupture surface or adjacent fault segments following the mainshock and is typically most pronounced during the early stages of the postseismic period before decaying rapidly with time [2,3]. In contrast, viscoelastic relaxation describes the time-dependent rheological response of the lower crust and upper mantle to earthquake-induced stress perturbations, generally producing regional deformation over substantially longer timescales [4]. Poroelastic rebound, which results from the redistribution of pore-fluid pressure following an earthquake, is generally characterized by a much smaller deformation signal and is difficult to isolate reliably using geodetic observations. Consequently, it is commonly neglected in long-term investigations of postseismic deformation [5,6,7].

Characterizing the temporal evolution of postseismic deformation is fundamental not only to understanding earthquake-induced transient deformation, but also to advancing our knowledge of long-term tectonic deformation and the earthquake cycle. Time-dependent deformation signals that persist after an earthquake can significantly influence the interpretation of present-day crustal motions and introduce substantial uncertainties into the estimation of interseismic velocity fields. Consequently, identifying and accounting for transient deformation associated with coseismic and postseismic processes is essential for deriving reliable crustal velocity fields and accurately quantifying strain accumulation. Beyond improving our understanding of lithospheric rheology, stress redistribution, and earthquake cycle dynamics, characterizing postseismic deformation also provides a critical foundation for seismic hazard assessment, crustal strain accumulation models, and earthquake probability estimates [8,9,10].

Global Navigation Satellite System (GNSS) observations have become one of the most reliable and widely adopted geodetic techniques for monitoring crustal deformation with millimeter-level precision [11]. By enabling precise measurements throughout the earthquake cycle, GNSS provides a powerful framework for quantifying interseismic strain accumulation, coseismic displacements, and postseismic transient deformation. Since their widespread deployment in the late 1990s, continuous GNSS (cGNSS) networks have transformed crustal deformation monitoring by providing uninterrupted observations of the earthquake cycle and have become indispensable for resolving the rapidly evolving deformation that characterizes the early postseismic period [12,13]. The high temporal resolution of continuous GNSS observations enables detailed modeling of the temporal evolution of postseismic deformation through logarithmic, exponential, and combined time-dependent functions fitted to GNSS time series. Such models facilitate the identification of distinct postseismic deformation signatures, allowing characteristic relaxation timescales and the temporal evolution of postseismic processes throughout the earthquake cycle to be quantified. Consequently, cGNSS networks have been extensively used to characterize postseismic transient deformation following numerous large earthquakes across a wide range of tectonic environments [14,15,16]. Despite the exceptional temporal resolution provided by continuous GNSS (cGNSS) networks, the spatial distribution of geodetic stations remains a critical factor in reliably characterizing postseismic deformation fields. In practice, establishing dense cGNSS networks is often constrained by topographic conditions, infrastructure availability, installation and maintenance costs, and the technical challenges associated with ensuring long-term data continuity. Consequently, sufficiently dense station coverage is not always achievable, particularly in the aftermath of large earthquakes, limiting the regional characterization of postseismic deformation. Under such circumstances, campaign GNSS observations continue to play an important role in monitoring postseismic deformation, as they do throughout other stages of the earthquake cycle. Although the sparse and irregular temporal sampling of campaign GNSS observations hampers the resolution of early postseismic transients and complicates the separation of coseismic, interseismic, and postseismic deformation signals, previous studies have demonstrated that campaign GNSS time series can successfully capture and model postseismic deformation [17,18,19]. Moreover, campaign observations spanning multi-year periods offer a distinct advantage by capturing the cumulative effects of postseismic deformation despite their relatively low temporal resolution, making this approach particularly effective when characteristic relaxation timescales extend from months to years.

In this context, the western Anatolian deformation zone, which has evolved under the complex interactions of the Arabian, Eurasian, and African plates, is recognized as one of the world’s most active extensional tectonic provinces [20]. In particular, Western Anatolia and the Aegean region, spanning the Türkiye–Greece plate boundary, constitute a natural laboratory for investigating crustal deformation owing to their high seismicity, active graben systems, and frequent occurrence of large earthquakes [21]. Within this tectonic setting, the Mw 7.0 Samos earthquake of 30 October 2020 generated a broad deformation field across the Eastern Aegean and Western Anatolia, producing coseismic displacements of up to approximately 40 cm near the epicenter and significant ground deformation around İzmir, where the earthquake caused the collapse of more than ten buildings along the coastal area [22,23]. Source models indicate that the earthquake ruptured a north-dipping normal fault, with unilateral westward rupture propagation and maximum slip concentrated west of the hypocenter [24,25]. Beyond the rupture geometry, studies based on Coulomb stress transfer and aftershock distributions suggest that the effects of the earthquake were not confined to the main rupture plane but may also have influenced adjacent transfer faults and neighboring fault segments [26,27,28]. Nevertheless, previous investigations of the Samos earthquake have primarily focused on coseismic deformation and the early stages of postseismic deformation, e.g., [24] whereas the long-term temporal evolution of postseismic deformation and the potential of campaign GNSS observations to characterize these transient processes remain largely unexplored.

Building upon these motivations, this study provides new geodetic insights into the long-term temporal evolution of postseismic deformation following the 2020 Mw 7.0 Samos earthquake by systematically integrating campaign GNSS observations with pre-earthquake velocity constraints and an AICc-based model-selection framework. The temporal evolution of postseismic deformation in and around İzmir was investigated using campaign and continuous GNSS observations from 18 stations spanning approximately 4.5 years. Pre-earthquake GNSS velocities were incorporated as prior constraints to reduce trade-offs between postseismic amplitudes and secular velocity parameters. Linear, logarithmic, exponential, and combined logarithmic–exponential functions were evaluated, and the preferred model for each station-component pair was selected using AICc. Characteristic-timescale uncertainty was represented using AICc support intervals, and model-selection stability was assessed through 1000 Monte Carlo observation–perturbation simulations. This framework provides new data-driven constraints on the spatial and temporal evolution of postseismic deformation across Western Anatolia while explicitly accounting for the limitations imposed by sparse campaign sampling.

## 2. Tectonic and Seismotectonic Setting

### 2.1. Regional Tectonic Framework

**Figure 1:**
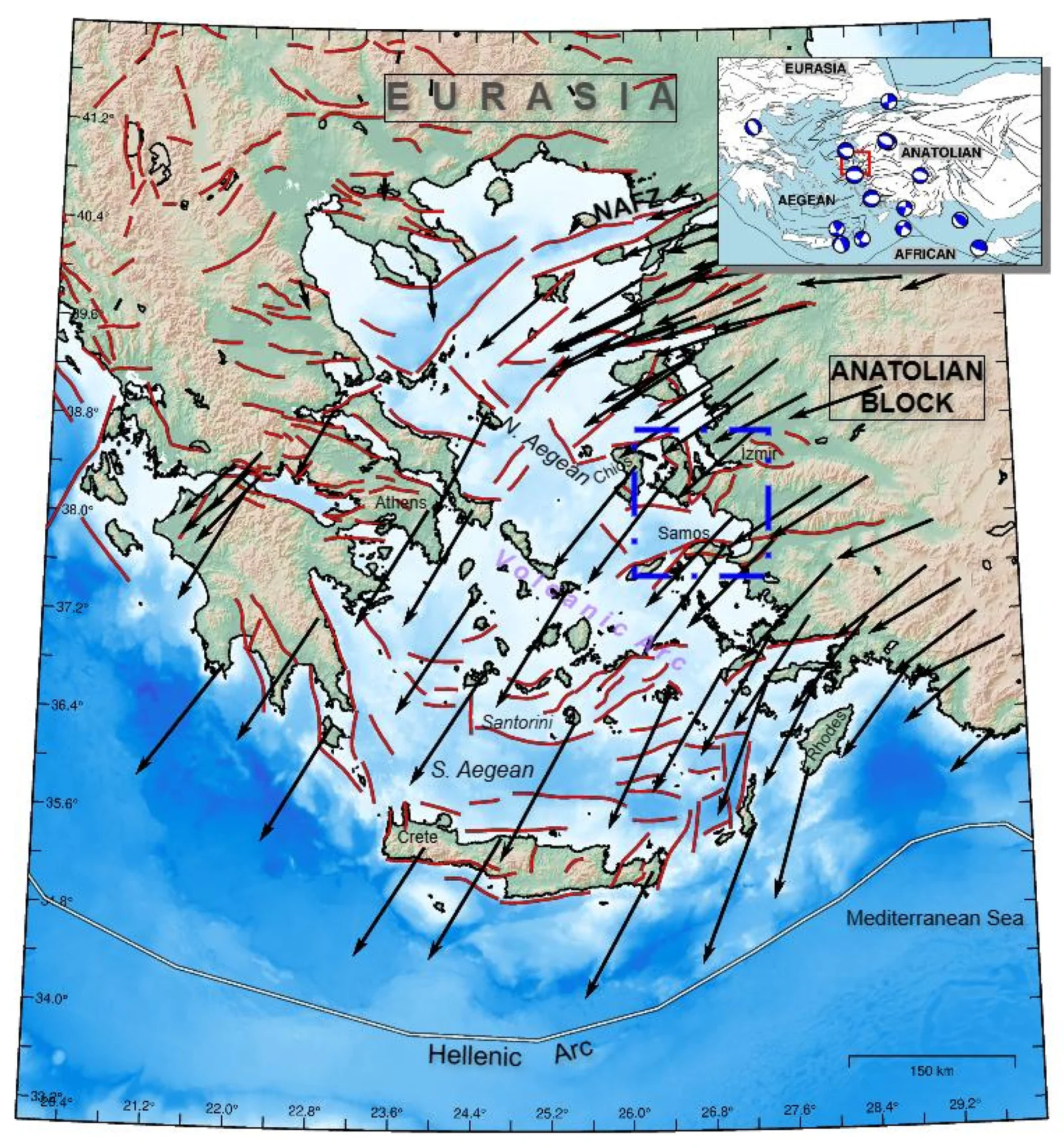
Regional tectonic setting of Western Anatolia and the Aegean region. Red lines indicate active fault systems compiled from [37,38,39,40,41,42,43,44,45,46,47,48,49,50], Blue dashed box denote study area and black arrows show the GNSS velocity field derived from [51] and [29]. The inset map shows the broader plate-tectonic setting of the Aegean region and the focal mechanism solutions in the area.

The Anatolian Plate is bounded by the North Anatolian Fault Zone (NAFZ) to the north and the East Anatolian Fault Zone (EAFZ) to the east, and undergoes westward extrusion, giving rise to a pronounced extensional regime across the Aegean region [22,24,29,30,31,32]. The Eastern Mediterranean, as an integral component of this extensional system, constitutes a complex deformational domain governed by the convergence between the African and Eurasian plates and the westward escape of the Anatolian Block. Western Anatolia and the Aegean Sea have been characterized by north–south (N-S) lithospheric extension since the Late Oligocene to Early Miocene (Figure 1). This extensional regime is widely attributed to rollback of the Hellenic subduction zone and has led to the development of a series of east–west (E-W)-trending graben systems throughout the region [33,34]. GNSS observations indicate that the Aegean region moves southwestward (SW) relative to the Eurasian Plate at rates of approximately 30–35 mm yr^−1^, with velocities increasing progressively toward the south [29,35,36].

Despite the regional extensional regime, parts of Western Anatolia, particularly the İzmir region, exhibit a considerably more complex deformational structure than can be explained by graben tectonics alone. The study area is located within the İzmir–Balıkesir Transfer Zone (IBTZ), a major tectonic transition zone characterized by NE-SW-trending strike-slip faults that intersect the E-W-trending normal fault systems of Western Anatolia [30,52,53]. The interaction between extensional and strike-slip deformation within this transfer zone is considered a primary driver of the pronounced spatial heterogeneity of crustal deformation and the elevated seismic hazard across the region.

Within this tectonic framework, numerous active faults with lengths ranging from approximately 10 to 50 km have been identified in and around İzmir. These structures can be broadly classified into two principal fault systems: (i) E-W-trending normal faults formed under the regional N-S extensional regime and (ii) strike-slip transfer faults that dissect and offset these extensional structures [33]. The coexistence of these fault systems indicates that crustal deformation is partitioned among multiple interacting structures rather than being accommodated by a single fault segment. Such structural complexity complicates the interpretation of postseismic deformation following large earthquakes and suggests that the observed deformation may reflect not only viscoelastic relaxation but also afterslip and triggered fault slip distributed across multiple fault segments.

**Figure 2:**
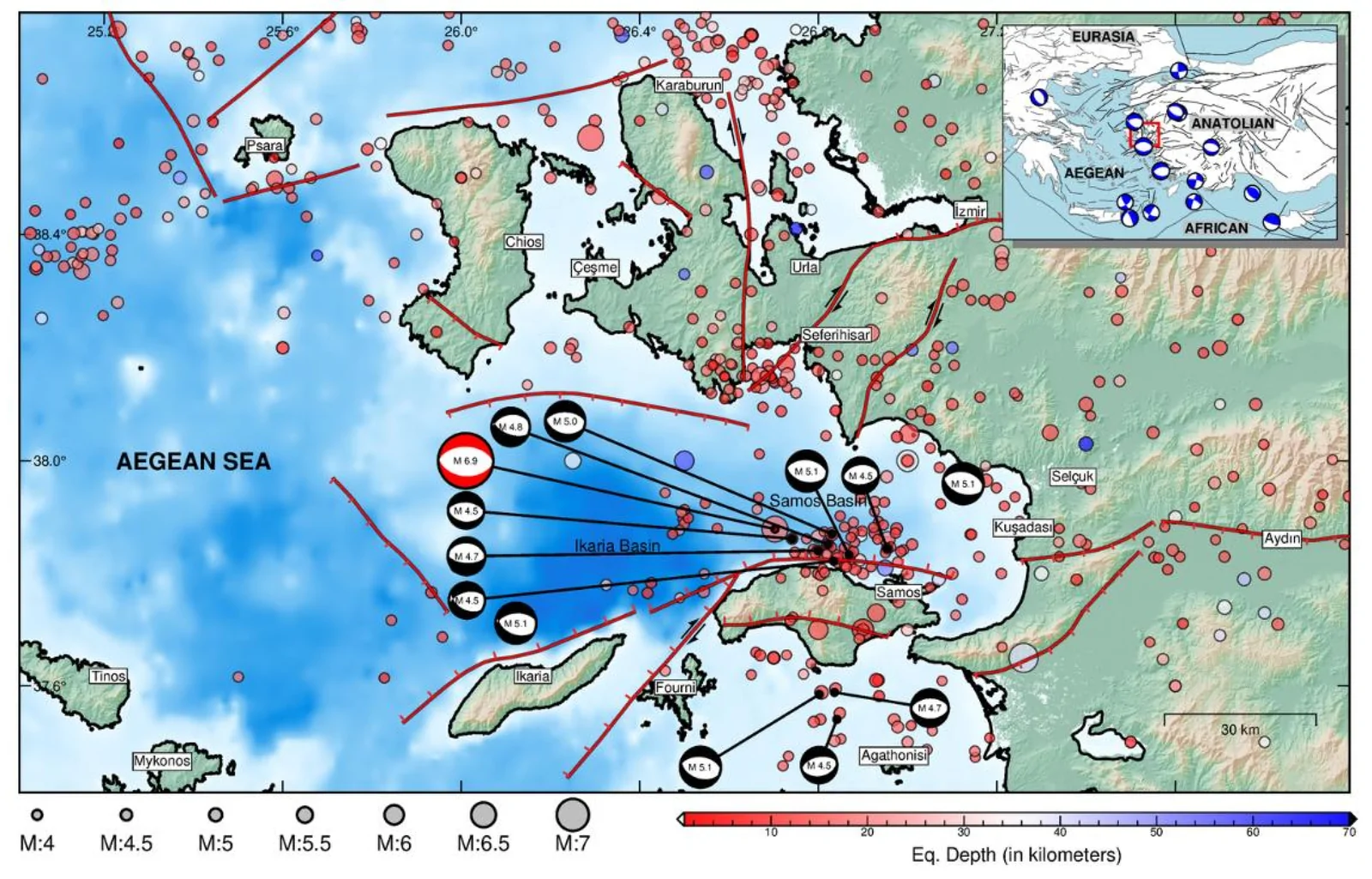
Local seismotectonic setting and focal-mechanism solutions of the 2020 Mw 7.0 Samos earthquake and the surrounding İzmir–Samos region. Red lines denote active fault structures [37,38,39,40,41,42,43,44,45,46,47,48,49,50], while circles show earthquake epicenters, with symbol size representing earthquake magnitude and color indicating focal depth.

Western Anatolia, which hosts this complex network of interacting tectonic structures, is recognized as one of the most seismically active and rapidly extending continental regions in the world [54]. Its high seismic potential is evidenced by numerous destructive earthquakes throughout the historical period and has remained equally pronounced during the instrumental era, with seismic activity continuously manifested by earthquakes spanning a wide range of magnitudes. As the completeness and quality of earthquake catalogues have improved, historical records dating back to the mid-nineteenth century document numerous devastating earthquakes with intensities ranging from VI to X, highlighting the persistent seismic hazard of the region [55,56]. Although seismicity in Western Anatolia and the Eastern Aegean is predominantly characterized by swarm-type earthquake activity [56], the region has repeatedly generated moderate-to-large earthquakes (Mw > 6) across the Aegean Sea, Western Anatolia, and offshore Greece, reflecting its complex deformational regime and high seismotectonic activity (Figure 2). In addition to thousands of earthquakes with M ≥ 3, several major events have occurred during the instrumental period, including the 1992 Doğanbey (Mw 6.0), 1999 West Aegean Sea (Md 6.0), 2003 Seferihisar (Mw 5.7), 2005 Sığacık earthquake sequence (Mw 5.7, 5.7 and 5.2), the 2017 Biga Peninsula shallow earthquake swarm (M > 5), the 2017 Karaburun (Mw 6.2), the 2017 Muğla (Mw 6.5), and most recently the 2020 Mw 7.0 Samos earthquake, which struck offshore of Western Anatolia [57].

### 2.2. Seismotectonic Characteristics of the 30 October 2020 Samos Earthquake

The 30 October 2020 Samos earthquake represents a typical appearance of the extensional tectonic regime in the Eastern Aegean and ruptured an E-W-trending, north-dipping normal fault located north of Samos Island [22,58]. The moment magnitude of the earthquake was reported between Mw 6.6 and Mw 7.2 by different seismological agencies (USGS, KOERI, AFAD, GFZ, and OCA), while focal mechanism solutions consistently indicate rupture on a moderately dipping (~37–45°) normal fault. Joint analyses of seismological and geodetic observations further demonstrate that the earthquake ruptured a single fault plane and propagated unilaterally toward the west [25]. Slip inversion models indicate that the maximum coseismic slip, reaching approximately 2.0–2.5 m, was concentrated west of the hypocenter at depths of about 10–15 km [24,26].

The mainshock occurred at a shallow depth and was assigned a maximum intensity of VIII (NOAA). It was felt over a broad region extending from the eastern Aegean to mainland Greece, including Athens and Heraklion, located nearly 300 km from the epicenter. Despite occurring offshore of Samos Island, the earthquake caused its most severe damage along the İzmir coastline, resulting in more than 100 fatalities, over 1000 injuries, and widespread structural damage [59,60]. The earthquake also generated a tsunami that caused an additional fatality on the Turkish coast [61]. During the first week following the mainshock, more than 2000 aftershocks were recorded, the largest reaching Mw 5.1. This intense aftershock sequence was primarily concentrated northeast of the main rupture and around its western termination, forming distinct clusters that delineate the activated fault system (Figure 2).

Coulomb stress-transfer analyses and aftershock-distribution studies conducted following the earthquake indicate that rupture effects were not confined to the main fault plane but also induced significant stress perturbations on surrounding fault systems [27,28,62,63,64]. Positive Coulomb stress changes at the eastern and western rupture terminations suggest that several active fault systems, particularly those beneath the İzmir Gulf and the Karaburun Peninsula, were brought closer to failure. Similarly, static stress-change calculations by [26] indicate that active faults around the Sığacık and Kuşadası bays experienced stress increases sufficient to promote future rupture. Beyond the Coulomb stress patterns, the spatial distribution of aftershocks suggests that deformation was not restricted to the main rupture plane but likely involved oblique and strike-slip transfer faults surrounding the source region [27,28]. Collectively, these observations indicate that deformation associated with the Samos earthquake cannot be fully explained by slip on a single fault plane alone, highlighting the importance of regional fault interactions when interpreting both coseismic and postseismic deformation.

Although previous studies of the Samos earthquake have primarily focused on coseismic deformation and short-term postseismic processes, the long-term temporal evolution of postseismic deformation remains poorly constrained from a geodetic perspective. In tectonically complex settings such as the İzmir region, where multiple interacting fault systems coexist, long-term monitoring of postseismic transient deformation is important not only for distinguishing the relative contributions of afterslip and viscoelastic relaxation, but also for evaluating stress loading on neighboring fault segments brought closer to failure by Coulomb stress transfer and assessing their implications for potentially triggered seismicity. In this context, regional-scale geodetic monitoring based on repeated GNSS observations with adequate spatial coverage provides the observational framework required to quantitatively characterize the long-term spatiotemporal evolution of postseismic deformation in such tectonically complex environments.

## 3. Data and Methodology

### 3.1. GNSS Network and Observations

**Figure 3:**
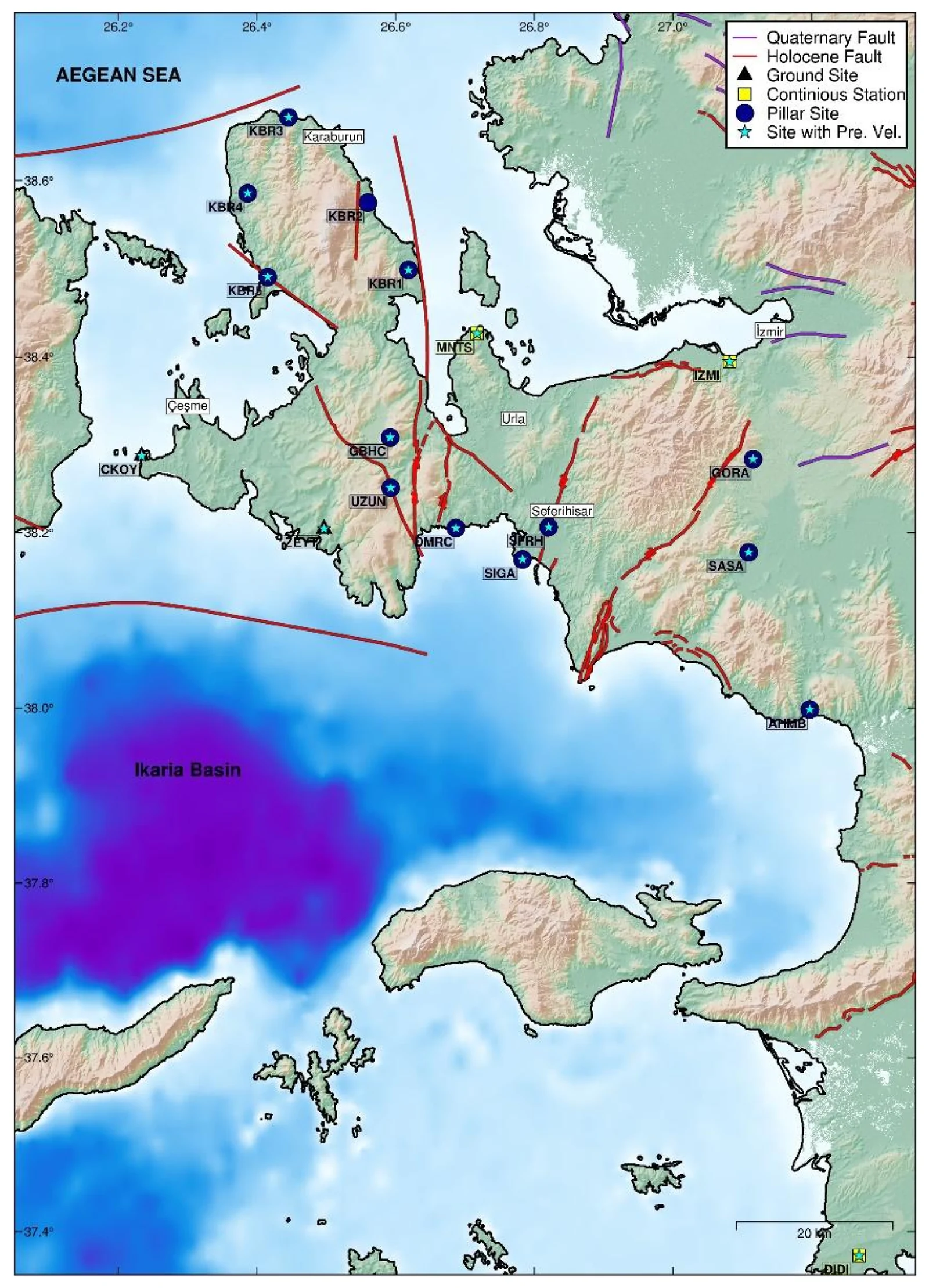
GNSS network used to characterize postseismic deformation following the 2020 Samos earthquake. Fault traces [49] are shown together with the locations of the campaign and continuous GNSS stations. Different symbols distinguish pillar sites, ground sites, and continuous GNSS stations, while station names are labeled adjacent to the corresponding sites.

To investigate the spatial and temporal evolution of postseismic deformation in and around İzmir, Western Anatolia, following the 30 October 2020 Mw 7.0 Samos (Aegean Sea) earthquake, a geodetic dataset comprising both campaign and continuous GNSS observations was analyzed. The GNSS network consists of 18 stations distributed across the İzmir, Karaburun, Çeşme, and Seferihisar regions (Figure 3), including 15 campaign stations and three continuous GNSS (cGNSS) stations.

Between 2020 and 2025, each campaign site was occupied during five to eight survey campaigns. Postseismic observations were available at 17 of the 18 stations within the first 10 days following the mainshock, providing valuable sensitivity to the early stage of postseismic deformation. At KBR2, the only late-start station, the first postseismic observation was acquired approximately 231.5 days after the earthquake. To maximize data quality and positioning precision, observation strategies were tailored according to station type. At pillar monuments, GNSS observations were collected during a single day in each campaign with a minimum observation duration of 8 h. At ground monuments, observations were conducted over two consecutive days with a minimum of 8 h of data acquisition per day to improve measurement reliability. In addition, chained tripods were used instead of conventional surveying tripods at ground monuments to enhance instrument setup stability and reduce height-related issues throughout the observation sessions.

### 3.2. GNSS Data Processing Strategy

The GNSS observations were processed using the GAMIT/GLOBK (version 10.71) scientific software package developed by the Massachusetts Institute of Technology (MIT) and the Scripps Institution of Oceanography (SIO), following the precise positioning procedures recommended by the International GNSS Service (IGS) [65]. In the first stage of the two-step processing strategy, daily GNSS phase observations were processed in the GAMIT module using the double-difference approach, while IGS final precise orbit products and Earth Orientation Parameters (EOPs) were adopted for satellite orbit and Earth rotation modeling. Ionospheric delays were eliminated using the first-order ionosphere-free (L3) linear combination, whereas tropospheric delays were modeled using the Vienna Mapping Function 1 (VMF1) together with zenith wet delay (ZWD) estimates at 2 h intervals [66]. Ocean tide loading corrections were applied using the FES2004 model, solid Earth tide effects were modeled in accordance with the IERS 2010 conventions, and receiver and satellite antenna phase center variations (PCVs) were corrected using absolute antenna calibration models. These processing steps yielded daily loosely constrained station coordinates and their associated covariance matrices (h-files) for subsequent network adjustment.

In the second stage of data processing, the daily loosely constrained solutions generated by GAMIT were combined with the corresponding IGS reference station solutions within the GLOBK module using a Kalman filtering approach. To generate station coordinate time series and estimate pre-earthquake station velocities, 25 IGS reference stations were incorporated into the network adjustment, thereby providing a robust connection to the global reference frame. Station coordinates were initially realized in the International Terrestrial Reference Frame 2014 (ITRF2014) using the No-Net-Rotation (NNR) stabilization strategy [67].

### 3.3. Time-Series and Data Conditioning

Robust modeling of postseismic deformation requires GNSS coordinate time series to be appropriately conditioned prior to analysis. Accordingly, the raw coordinate solutions were transformed into relative displacements, artificial weighting effects associated with irregular observation intervals were mitigated, unrealistically small coordinate uncertainties were regularized, and the influence of outlying observations on the model solution was minimized. Together, these preprocessing steps established a consistent analysis framework that enabled campaign and continuous GNSS observations, despite their markedly different temporal sampling characteristics and data quality, to be modeled within a unified framework.

Following preprocessing, station coordinate solutions were transformed into relative displacement time series by referencing all observations to a pre-earthquake reference position. For each station component, the reference coordinate was defined using the latest available pre-earthquake epochs. When three or more pre-earthquake observations were available, only the last three epochs were used to establish the reference position; otherwise, all available pre-earthquake observations were included. The reference coordinate and corresponding reference epoch were computed using inverse-variance weighting, and all subsequent displacements were expressed in millimeters.

For campaign sites, consecutive observations separated by less than 7 days were combined into a single composite epoch using inverse-variance weighting. This procedure represented closely spaced sessions belonging to the same campaign period by a single temporally representative observation and prevented them from being interpreted as separate stages of postseismic evolution. The corresponding uncertainty was calculated using Equation (1), where the weight of observation *i* was defined as w_*i* = 1/σ_*i*^2^.

1/√(∑w*i*) (1)

To prevent unrealistically small formal uncertainties from disproportionately influencing the model solution, minimum uncertainty thresholds were imposed. Threshold values of 1.5 mm and 0.5 mm were adopted for campaign and continuous GNSS observations, respectively, and any smaller formal uncertainties were replaced by the corresponding threshold value. This distinction reflects the inherently lower repeatability of campaign observations, which are more susceptible to setup-related or antenna-height uncertainties, shorter observation sessions, and environmental effects than continuously operating GNSS stations.

Pre-earthquake velocity estimates were incorporated into the model as soft constraints through a Gaussian prior. The prior was implemented by extending the objective function with an additional residual term, defined as

(2) $$r_{v}=\frac{v-v_{0}}{\sigma_{v_{0}}}$$

where *v* denotes the model-estimated secular velocity, *v*_0_ is the independently estimated pre-earthquake velocity, and *σ_v_*_0_ represents its associated standard error. The prior residual was treated as an additional observation in both the residual vector and the likelihood function, thereby ensuring that the covariance matrix and the reduced chi-square ($\chi_{red}^{2}$) statistics were evaluated consistently under the imposed prior constraint.

Outlier detection followed a conservative down-weighting strategy in which potentially problematic observations were identified without being automatically discarded from the dataset. Instead, their influence on the model solution was reduced. For each station-component time series, only interior epochs were evaluated. The normalized deviation of each observation was computed relative to the value predicted by linear interpolation between its adjacent epochs. An observation was flagged as a potential outlier only if three criteria were simultaneously satisfied: (i) the normalized deviation exceeded the 5σ threshold, (ii) the temporal gap between the neighboring epochs was less than 540 days, and (iii) the observation exhibited a local sign reversal. To avoid excessive intervention, no more than one observation was flagged within each station-component time series. Flagged observations were retained in the dataset but assigned reduced weights by inflating their standard deviations by a factor of four. Because observation weights were defined as

(3) $$w=1/\sigma^{2}$$

This adjustment reduced the weight of the corresponding observation to 1/16 of its original value. This strategy follows the fundamental principle of robust estimation, whereby observations associated with large residuals are down-weighted rather than discarded, thereby limiting their influence on the model solution [68,69,70,71]. The inflation factor of four was not intended as a universal threshold but was adopted as a conservative weighting parameter that substantially reduced the influence of potentially problematic observations while retaining them in the estimation process.

Observations acquired within the first 120 days following the mainshock were excluded from the outlier screening procedure, as rapid transient deformation during this interval may represent the exact postseismic signals rather than observation anomalies. Following these conditioning procedures, the campaign and continuous GNSS datasets, despite their heterogeneous temporal sampling and uncertainty characteristics, were integrated into a consistent weighted dataset with systematic biases mitigated and pre-earthquake velocity information incorporated through soft constraints. This conditioned solid dataset provided the basis for the model selection and parameter estimation procedures described in the following sections.

### 3.4. Postseismic Deformation Modeling

The temporal evolution of postseismic deformation can be represented using different functional forms depending on the dominant deformation processes and the rheological properties of the lithosphere. Logarithmic decay functions are commonly used to approximate rapidly decaying afterslip-like behavior, whereas exponential functions are often used to represent longer-timescale relaxation [4,72]. Combinations of these functions can represent time series containing more than one temporal component. These functions are empirical descriptions of temporal behavior and do not uniquely identify the underlying physical mechanism. In this study, the alternative postseismic models were therefore evaluated independently for each station-component time series within an objective model-selection framework.

Modeling postseismic deformation from campaign GNSS observations presents several statistical and physical challenges. GNSS time series are affected by both white and temporally correlated noise [73,74], while strong trade-offs commonly exist between postseismic amplitudes and the characteristic relaxation time (τ) [75]. In sparsely sampled datasets such as campaign-style GNSS observations, spectral overlap between seasonal signals and postseismic transients may further bias amplitude estimates. Moreover, when only limited pre-earthquake observations are available, simultaneously estimating the interseismic velocity introduces additional uncertainty that directly propagates into the postseismic model parameters. Therefore, robust model selection and rigorous uncertainty quantification are particularly important when analyzing campaign GNSS time series. Accordingly, the modeling framework adopted in this study places particular emphasis on objective model selection and robust uncertainty assessment.

Postseismic deformation was modeled independently for each station-component time series using four candidate functional forms: linear (LIN), logarithmic (LOG), exponential (EXP), and combined logarithmic-exponential (LOGEXP) models. All models were referenced to the earthquake origin time (t0), and the postseismic terms were defined only for epochs satisfying t ≥ t0. The linear model represents the secular deformation component, whereas the LOG, EXP, and LOGEXP models additionally incorporate time-dependent transient deformation following the earthquake. The four candidate models considered in this study are expressed as follows:

(4) $$m(t)\;=\;a\;+\;v(t\;-\;t_{ref})\;+\;C\;H(t\;-\;t_{0})\;+\;P(t)$$

where a denotes the constant position offset, v is the secular velocity, C represents the coseismic offset, H(t − t0) is the Heaviside step function, and P(t) denotes the postseismic transient term. For the linear model, P(t) is set to zero. The logarithmic and exponential transient functions are respectively defined as follows. All time variables used in the model equations were expressed in decimal years; reported relaxation times were converted to days.

(5) $$P_{LOG}(t)=A_{log}\;ln(1+\frac{t-t_{0}}{\tau_{log}})$$

(6) $$P_{EXP}(t)=A_{exp}\;[1-exp(-\frac{t-t_{0}}{\tau_{exp}})]$$

For the LOG, EXP, and LOGEXP models, the characteristic relaxation time (τ) was selected from predefined discrete candidate grids rather than estimated as a continuous free parameter. This strategy reduced unstable and physically implausible solutions arising from the strong trade-off between postseismic amplitude and relaxation time. The campaign-specific relaxation-time grid was designed with consideration of the temporal sampling characteristics and overall observation span of the campaign GNSS dataset. For all campaign GNSS time series, the single-process LOG and EXP models were evaluated using the restricted grid:

τ = [182.6, 292.2, 438.3, 730.5] days

The selected values correspond to 0.5, 0.8, 1.2, and 2.0 years (182.6, 292.2, 438.3, and 730.5 days), thereby providing a set of intermediate- to long-term timescales that can be meaningfully constrained by the available campaign observations. These values were selected as practical candidate timescales spanning the temporal resolution of the campaign measurements and the multi-year duration of the dataset. For the three cGNSS stations, the single-process LOG and EXP models were evaluated using the extended grid:

τ = [14.6, 29.2, 58.4, 109.6, 182.6, 292.2, 438.3, 730.5] days

For the LOGEXP model, the logarithmic and exponential timescales were selected independently from separated candidate grids: τ_log = [14.6, 29.2, 58.4, 109.6] days and

τ_exp = [182.6, 292.2, 438.3, 730.5] days.

Station-component time series were classified as early-start (≤30 days) or late-start (>30 days) according to the interval between the earthquake and the first postseismic observation. This classification was used to identify series for which a coseismic offset could be separated from the early postseismic transient. The two KBR2 components were the only late-start time series.

Because of its higher parameter dimensionality, the combined LOGEXP model was considered only for the three cGNSS stations and was not included in the candidate-model set for campaign GNSS time series. The LOGEXP model introduces four postseismic parameters (A_log, A_exp, τ_log, and τ_exp), whose well-constrained estimation requires temporally dense observations spanning both the early and long-term stages of postseismic deformation. This restriction reduced parameter trade-offs and the risk of overparameterization. For early-start station-component time series, each candidate model was evaluated both with and without the coseismic offset parameter C, and the preferred configuration was selected using AICc. For the late-start KBR2 time series, C was not independently estimated because the absence of observations near the earthquake epoch prevents the coseismic step from being reliably separated from the early postseismic transient. Each GNSS observation is assumed to follow an independent Gaussian error model, εi ~ N(0, σi^2^), with the probability density function:

p(y_i_|θ) = (2πσ_i_^2^)^−1^/^2^ · exp(−(y_i_ − f(t_i_; θ))^2^/(2σ_i_^2^))

The data log-likelihood over ndata observations is

log L_data(θ) = −(1/2) · Σ_i_ [(y_i_ − f(t_i_; θ))^2^/σ_i_^2^ + log(2πσ_i_^2^)]

The pre-seismic velocity constraint is incorporated as an independent Gaussian prior, v ~ N(v0, σv0^2^), contributing an additional log-likelihood term:

log L_prior = −(1/2) · [(v − v_0_)^2^/σ_v0^2^ + log(2πσ_v0^2^)]

The total log-likelihood is log L = log Ldata + log Lprior, with effective observation count ntotal = ndata + 1. AICc is defined as

AICc = −2 log L + 2k + 2k(k + 1)/(n_total − k − 1)

where k denotes the number of free parameters, augmented by +1 for LOG and EXP models and +2 for LOGEXP to account for the effective model complexity introduced by the discrete τ grid search. The covariance matrix is scaled by χ^2^ red computed over the full ntotal-observation system, ensuring consistent propagation of the velocity prior into parameter uncertainties.

### 3.5. Model Selection and Derived Metrics

Model selection represents a critical component of postseismic deformation analysis because the adopted functional form directly governs both the estimated postseismic amplitudes and the characteristic relaxation times, thereby influencing the derived displacement metrics and their geophysical interpretation. This issue is particularly important for sparsely sampled campaign-style GNSS time series, where multiple competing models may provide statistically comparable fits to the observations. Under such conditions, model selection should account not only for goodness-of-fit but also for model complexity. Accordingly, the corrected AICc was adopted to balance model fit against model complexity. For each station-component time series, the candidate model structure, characteristic relaxation time (τ), and coseismic offset configuration were jointly optimized within a unified AICc-based model selection framework [76,77].

For AICc computation, the effective number of model parameters (k) accounted for the additional flexibility introduced through discrete relaxation-time selection. Accordingly, k included one additional parameter associated with τ selection for the LOG and EXP models and two additional parameters for the independent selection of τ_log and τ_exp in the LOGEXP model. This formulation ensured that the grid-search flexibility was represented consistently in the AICc complexity penalty.

Candidate solutions were first ranked according to their AICc values. When multiple candidates fell within ΔAICc < 2 of the minimum AICc, the final solution was selected according to parsimony by choosing the candidate with the fewest parameters; when parameter counts were equal, the candidate with the lower AICc was retained. The family-level support statistic, denoted ΔAICc_family, was calculated as the AICc difference between the selected model family and the best-performing alternative model family and was used for the very strong, strong, moderate, and ambiguous support classifications. Because τ was selected from a discrete grid rather than optimized continuously, a conventional covariance-based standard error was not assigned. Instead, τ uncertainty was represented by an AICc support interval calculated within the selected model family and coseismic-offset configuration. The minimum and maximum τ values among solutions satisfying ΔAICc < 2 defined the supported interval.

Using the preferred model for each station-component time series, postseismic and total displacement metrics were calculated at 0.5, 1.0, and 2.0 years after the earthquake. For late-start EXP solutions, the fraction of asymptotic postseismic deformation that occurred before the first observation was calculated as f_missed = 1 − exp(−d/τ), where d is the delay between the earthquake and the first postseismic observation. The complementary fraction, f_remaining = exp(−d/τ), represents the deformation remaining after the first observation epoch. Because the LOG and LOGEXP models do not predict a finite asymptotic displacement, this calculation was applied only to EXP solutions.

Although AICc provides a deterministic model selection for one realization of the observations, small perturbations may alter the preferred model in sparsely sampled time series. Model selection stability was assessed using parametric Monte Carlo simulation (*n* = 1000 iterations) rather than residual bootstrapping. In each iteration, every observation y_i_ is independently perturbed by a zero-mean Gaussian variate scaled to its individual measurement uncertainty σ_i_:

y_i_* = y_i_ + ε_i_*, ε_i_* ~ N(0, σ_i_^2^)

The epoch structure, per-observation sigma floors, velocity prior, and observation weights are held fixed across all iterations. The complete modelling pipeline is then applied identically to each perturbed realization, and the fraction of iterations selecting the same model family as the original solution is recorded as MC_best_pct. This approach was preferred over residual bootstrapping because the observation uncertainties are heterogeneous and individually estimated; drawing noise from a pooled residual distribution would misrepresent the true error structure, and resampling with replacement can artificially inflate the effective sample size in sparse campaign time series, distorting the AICc calculation.

### 3.6. Uncertainty and Quality Assessment

Parameter uncertainties and model reliability were evaluated jointly because estimation quality varies among station-component time series with observation density, data quality, and model complexity. Well-constrained time series typically yield reduced chi-square (χ^2^_red) values close to unity and weighted root-mean-square (WRMS) residuals below approximately 2 mm, whereas noisier or sparsely sampled series may yield substantially larger values. Uncertainties of derived postseismic quantities were propagated from the covariance matrix of the preferred model using standard error-propagation techniques. Because τ was selected through a discrete grid, its model-selection uncertainty was reported separately using the AICc-supported interval described in Section 3.5. For propagation to the derived displacement metrics, the effect of discrete τ sampling was additionally approximated using a grid-spacing-based uncertainty term. The velocity-prior term was incorporated consistently into χ^2^_red, the log-likelihood, and covariance calculations. Accordingly, the effective number of observations was defined as follows:

(7) $$n_{\mathrm{total}}=n_{\mathrm{data}}+1$$

where the additional observation represents the normalized velocity-prior residual. The total chi-square statistic therefore included contributions from both the data residuals and the normalized prior residual. Likewise, the parameter covariance matrix was scaled using the overall χ^2^_red, including the prior contribution, and n_total was used consistently in the AICc calculation. This formulation ensured that the pre-earthquake velocity constraint contributed consistently to model selection and parameter-uncertainty estimation.

Model performance was evaluated using χ^2^_red, the weighted root-mean-square residual (WRMS), and consistency between the estimated secular velocity and the pre-earthquake velocity prior. Velocity consistency was quantified as the difference between the estimated and prior velocities normalized by their combined standard uncertainty and expressed in standard-deviation units. Four quality flags were defined: (i) χ^2^_red > 3.0, (ii) WRMS > 5.0 mm, (iii) ΔAICc_family < 2, and (iv) velocity inconsistency exceeding 3σ. These flags were not automatic rejection criteria but identified solutions requiring additional caution during interpretation.

Monte Carlo simulations were used to evaluate the robustness of the AICc-selected model family with respect to observational uncertainty. For each station-component time series, MC_best_pct was defined as the proportion of successful realizations assigned to the most frequently selected model family. Model-family selection was classified as stable (MC_best_pct ≥ 80%), moderately stable (60–79%), or unstable (<60%). In the present analysis, the modal Monte Carlo model family was identical to the model family selected from the original observations for all 36 time series. Station-component time series with MC_best_pct < 60% were classified as unstable, and model-specific quantitative interpretations were avoided for these solutions.

To assess the potential influence of seasonal signals on campaign GNSS time series, annual and semiannual harmonic components were estimated from the three cGNSS stations (DIDI, IZMI, and MNTS). Estimated annual amplitudes ranged from 0.3 to 2.3 mm in the east component and from 0.7 to 2.3 mm in the north component. The peak-to-peak seasonal variation between the principal campaign epochs was approximately 1.9 mm for the east component and 2.8 mm for the north component. These amplitudes correspond to less than 20% of the expected postseismic signal in the east component and approximately 28% in the north component. Because campaign observations were concentrated mainly in November and April, unresolved seasonal motion may alias into the estimated transient signals rather than being independently separated. The harmonic analysis indicates that this contribution is generally of millimeter scale but may be relevant for station components with relatively small postseismic amplitudes.

## 4. Results

### 4.1. Characteristics of the GNSS Dataset and Deformation Signals

A total of 36 GNSS time series, comprising the east and north components of 18 stations, were analyzed. Epicentral distances ranged from 30.2 to 91.8 km, with a median distance of 54.6 km, providing near- and intermediate-field coverage. Of the 36 station-component time series, 34 were categorized as early-start and the two KBR2 components were classified as late-start. The first postseismic observation at KBR2 was acquired approximately 231.5 days after the mainshock. Only three observations were identified as potential outliers and were retained with reduced weights through variance inflation, indicating generally high internal consistency of the processed time series.

**Figure 4:**
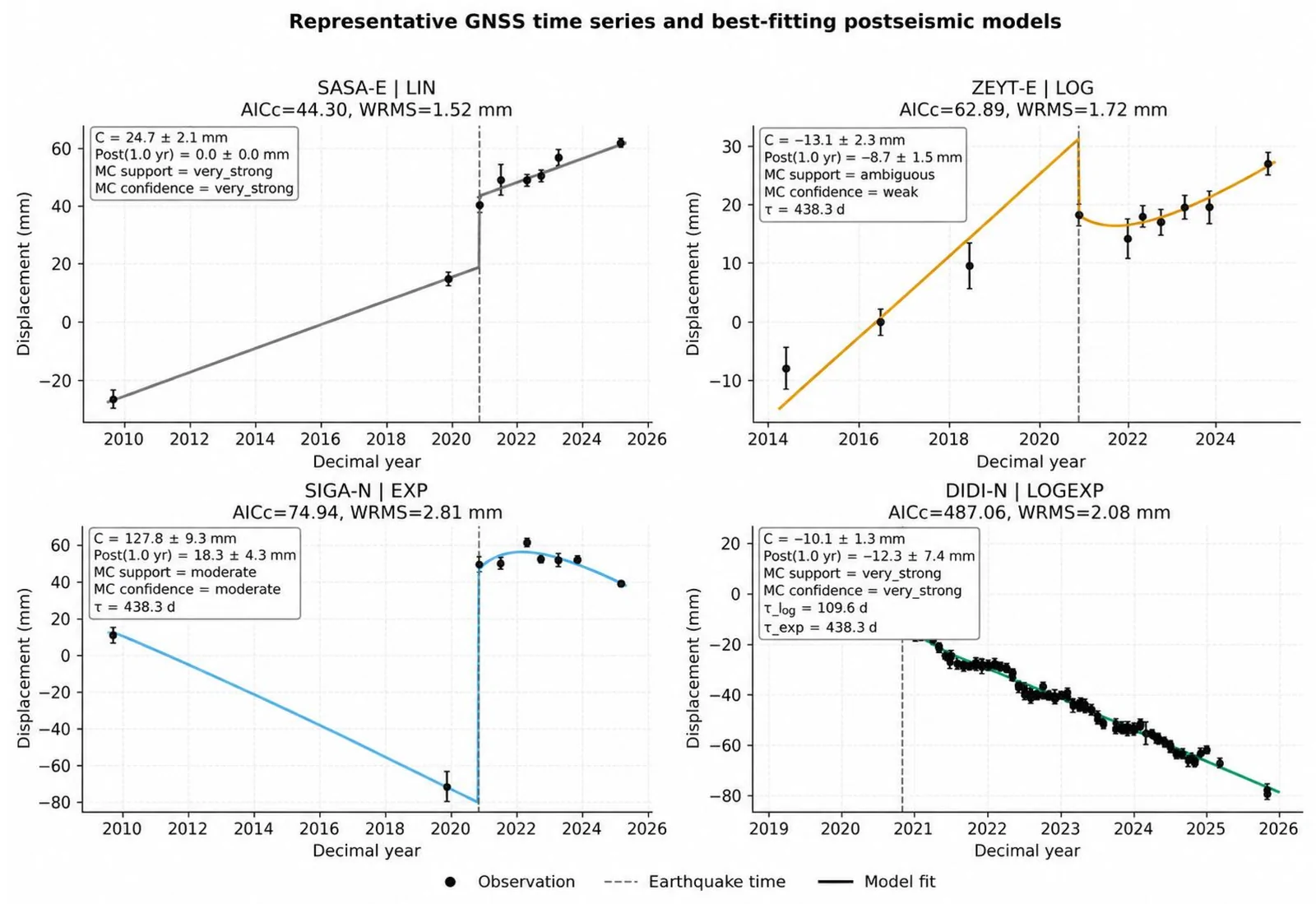
Representative GNSS time series and best-fitting postseismic models for selected stations. Black dots denote observations, dashed lines indicate the earthquake time, and solid lines show the preferred model fits.

Before modeling, campaign observations acquired within 7 days of one another were combined into a single weighted-mean epoch so that closely spaced sessions from the same campaign period were represented by one temporal observation. Prior to the earthquake, the GNSS time series exhibit only millimeter-level variability. Following this stable pre-earthquake behavior, the time series are characterized by distinct coseismic offsets followed by time-dependent postseismic deformation. Coseismic displacements vary considerably across the network, ranging from more than 100 mm at near-field stations to only a few millimeters at the most distant sites. Representative GNSS time series and their preferred postseismic models are presented in Figure 4.

### 4.2. Model Selection Performance and Stability

Model selection results reveal pronounced spatial variability in postseismic behavior across the GNSS network. Of the 36 station-component time series, 18 (50.0%) were best described by the linear (LIN) model, 9 (25.0%) by the logarithmic (LOG) model, 7 (19.4%) by the exponential (EXP) model, and 2 (5.6%) by the combined logarithmic–exponential (LOGEXP) model. The LOGEXP model was selected only for DIDI-N and IZMI-E, both of which are cGNSS time series, reflecting the greater temporal resolution required to constrain its additional parameters.

Family-level AICc support varied substantially among the analyzed time series. Thirteen time series (36.1%) exhibited very strong support for the selected model family, 5 (13.9%) strong support, and 7 (19.4%) moderate support, whereas 11 (30.6%) were classified as ambiguous (ΔAICc_family < 2.0). Most ambiguous solutions occurred in sparsely sampled campaign time series, where trade-offs between time-dependent transient deformation and the secular trend limited statistical discrimination among competing model families.

**Figure 5:**
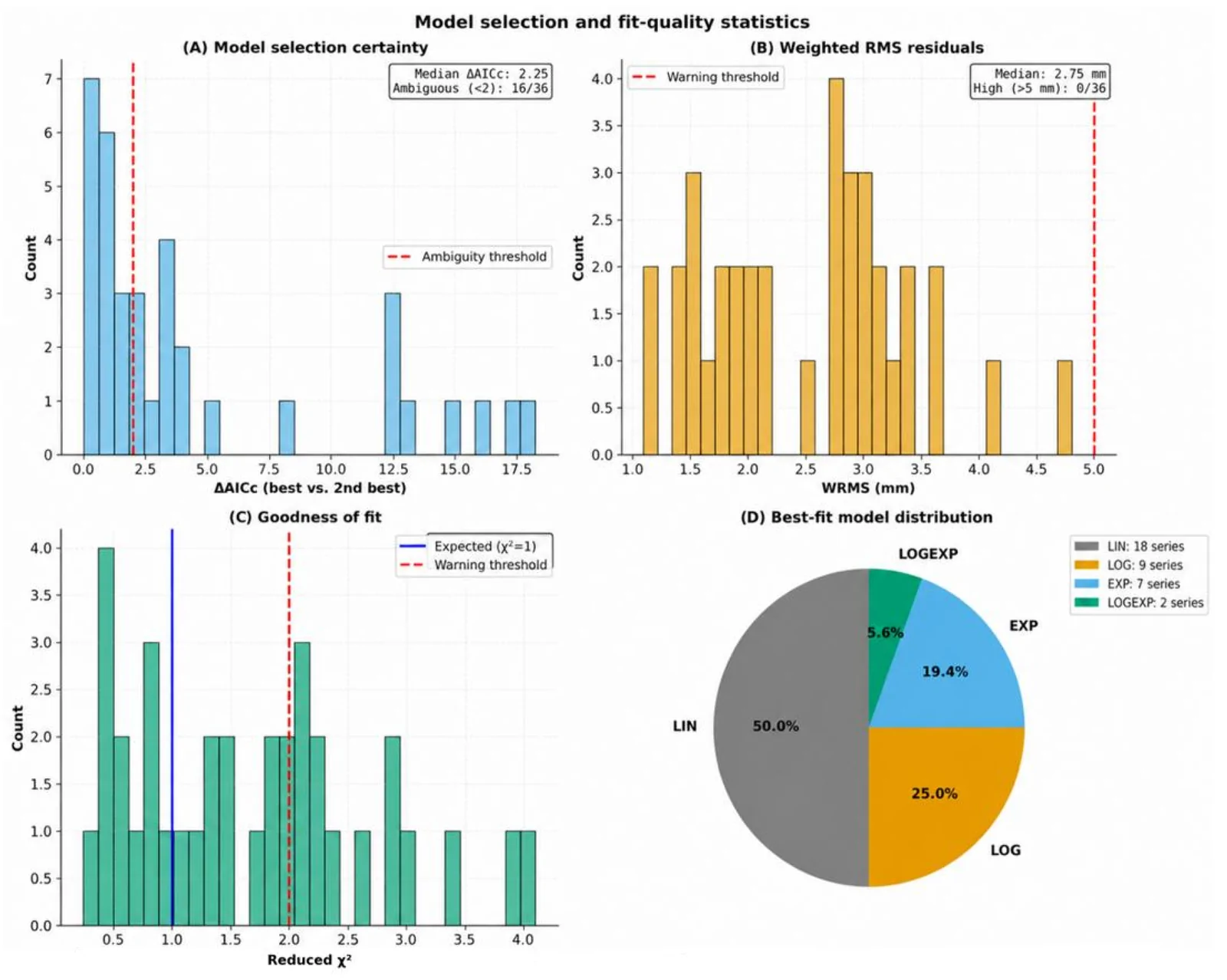
Model selection and fit-quality statistics including the distributions of ΔAICc, WRMS, reduced χ^2^, and preferred model types.

The 1000 Monte Carlo observation-perturbation simulations indicate that AICc-based model selection was generally stable with respect to the assigned observational uncertainties. Based on the predefined thresholds, 24 time series were classified as stable (MC_best_pct ≥ 80%), 10 as moderately stable (60–79%), and 2 as unstable (<60%). The unstable solutions corresponded to KBR1-N and KBR5-E, for which multiple model families were selected with comparable frequencies. Model-dependent quantitative interpretations were therefore avoided for these time series. The distributions of model-selection statistics and goodness-of-fit metrics are presented in Figure 5.

### 4.3. Coseismic Displacements and Early Postseismic Evolution

**Figure 6:**
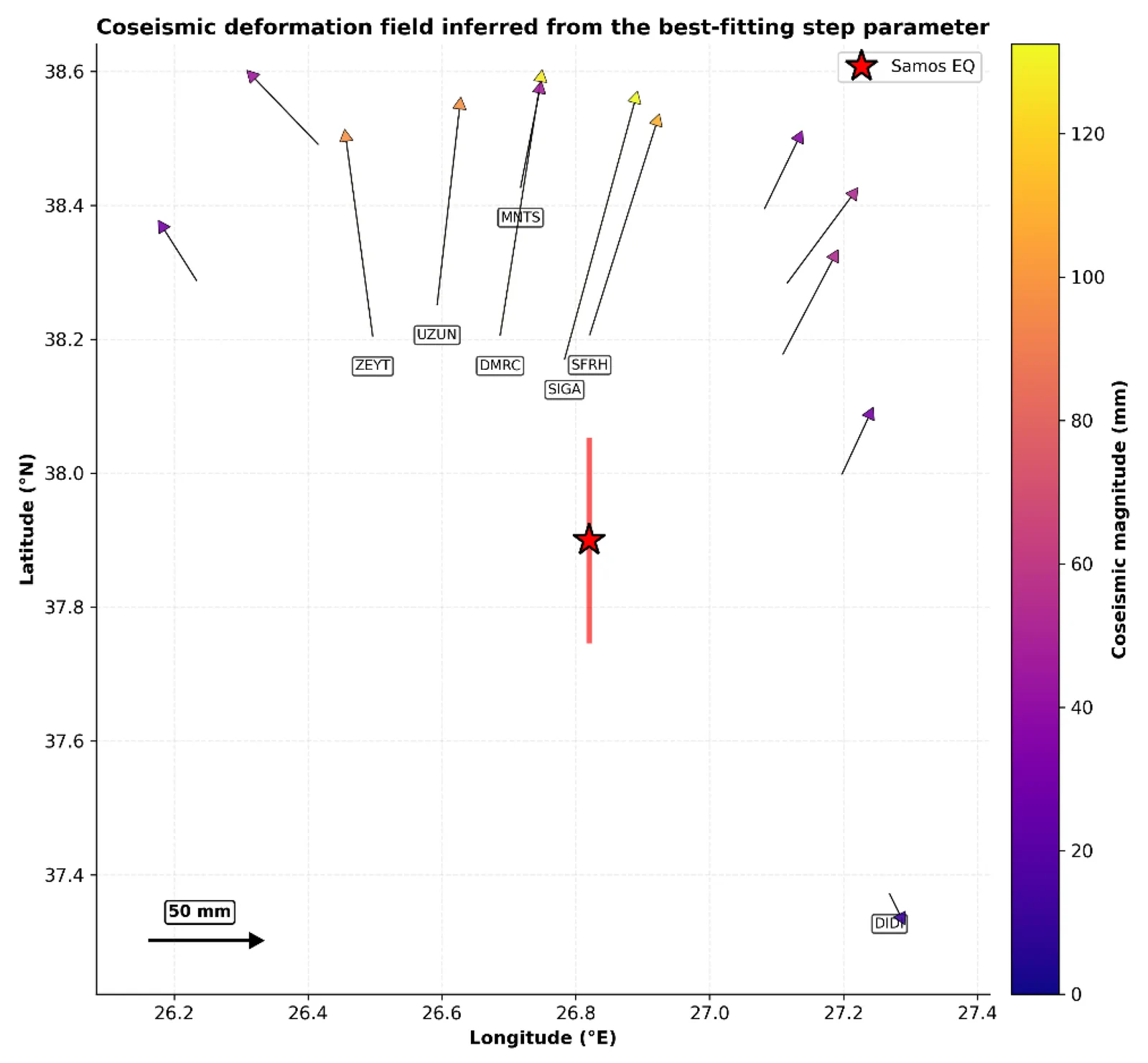
Coseismic horizontal displacement field inferred from the GNSS step parameter (C). Arrow directions indicate displacement vectors and colors represent displacement magnitude; the red star marks the Samos earthquake epicenter.

The estimated coseismic displacement parameter C exhibits pronounced spatial variability across the GNSS network. Among the 30 station-component time series for which a coseismic offset was selected, C ranged from −31.5 to +127.8 mm, with a median of 32.2 mm and a mean of 37.1 mm. The largest displacements (>100 mm) were observed at near-field stations located approximately 30–36 km from the epicenter (SIGA-N: 127.8 mm, DMRC-N: 126.5 mm, and SFRH-N: 104.4 mm). The coexistence of positive and negative components reflects the directional nature of the coseismic deformation field imposed by the fault geometry. The horizontal coseismic displacement field is shown in Figure 6.

Thirty-four of the 36 time series (94.4%) include observations acquired within the first 30 days following the earthquake and therefore directly sample the initial postseismic evolution. For the late-start KBR2 time series, the EXP models indicate that approximately 41.0% of the asymptotic postseismic deformation in the east component and 71.8% in the north component had occurred before the first observation epoch. Correspondingly, approximately 59.0% and 28.2% of the asymptotic deformation remained after the first observation in the east and north components, respectively. These results demonstrate that delayed campaign measurements may miss a substantial fraction of the early postseismic deformation.

### 4.4. Characteristics of Postseismic Deformation

**Figure 7:**
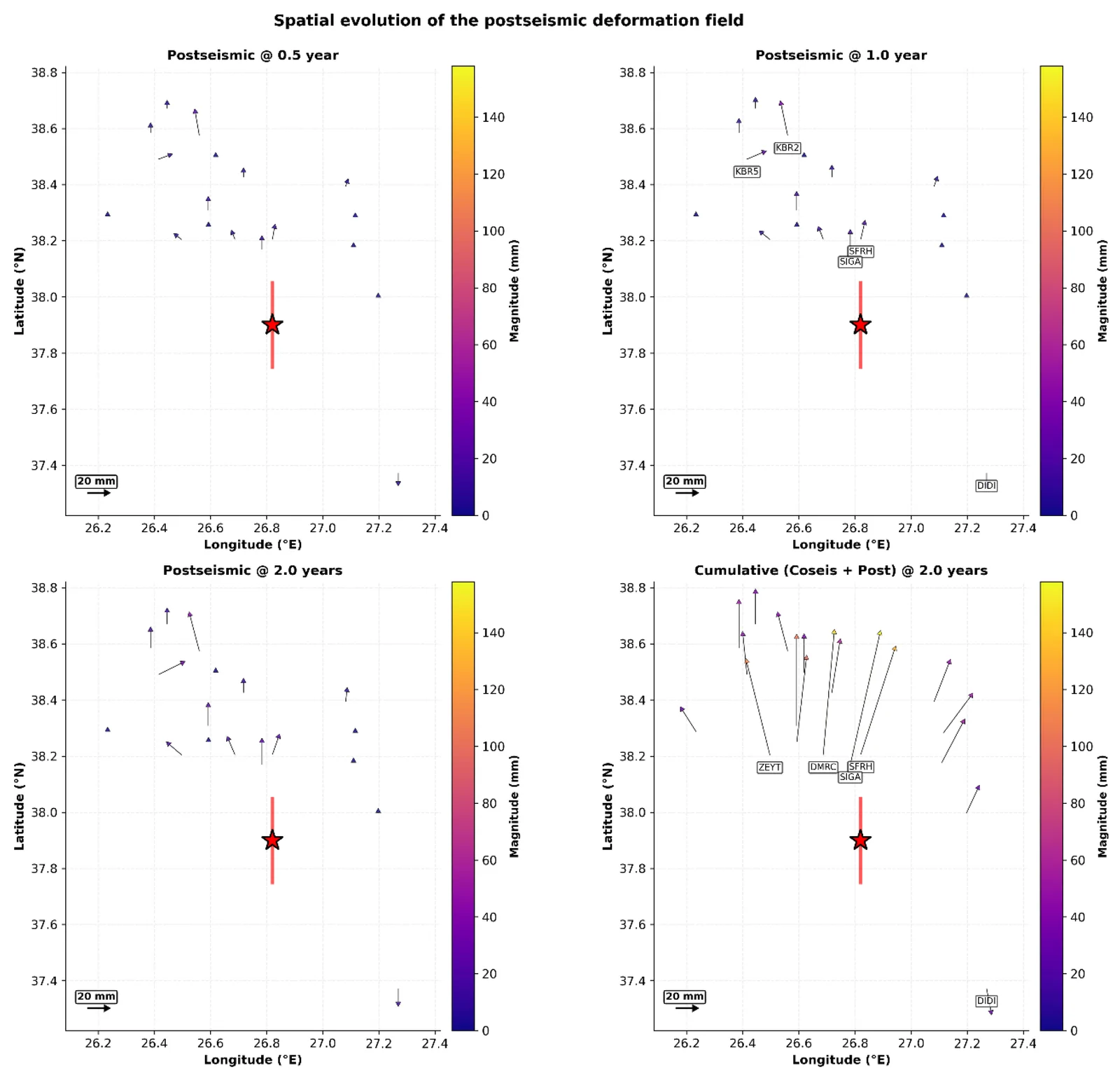
Spatial evolution of the postseismic deformation field at 0.5, 1.0, and 2.0 years after the earthquake, together with cumulative coseismic plus postseismic deformation. The red star marks the Samos earthquake epicenter.

The estimated postseismic signal exhibits pronounced spatial variability across the GNSS network (Figure 7). Postseismic displacements calculated at 0.5 years ranged from −9.9 to +26.4 mm and increased to a range of −12.3 to +36.2 mm at 1.0 year. Half of the analyzed time series were best represented by the LIN model, indicating that time-dependent transients could not be resolved with sufficient confidence under the available temporal sampling at those stations. Applying the predefined Monte Carlo and goodness-of-fit criteria (MC_best_pct ≥ 80% and χ^2^_red < 3.0) identified nine nonlinear station-component solutions that passed both filters (Table S1). Four were represented by EXP models (KBR2-E, KBR2-N, SFRH-N, and SIGA-N), three by LOG models (KBR3-N, KBR5-N, and ZEYT-N), and two by LOGEXP models (DIDI-N and IZMI-E). Although KBR5-N passed the Monte Carlo and goodness-of-fit filters, its family-level AICc support remained ambiguous; its model-specific interpretation should therefore be treated cautiously.

Among the seven single-process LOG and EXP solutions passing the Monte Carlo and goodness-of-fit filters, characteristic relaxation times ranged from 182.6 to 730.5 days, with a median of 438.3 days. Three solutions reached the upper boundary of the campaign τ grid at 730.5 days, whereas two reached the lower boundary at 182.6 days. Reaching a grid boundary indicates that the available observations do not distinguish timescales beyond the tested range and does not by itself demonstrate a longer or shorter true relaxation time [19,78]. The two LOGEXP solutions yielded a logarithmic timescale of 109.6 days and exponential timescales of 292.2 and 438.3 days. The KBR4-N time series was excluded from quantitative interpretation because of its poor model fit (χ^2^_red = 4.10, WRMS = 4.81 mm). Although its Monte Carlo stability was 100%, the elevated misfit indicates that the parameter estimates are not sufficiently reliable for quantitative interpretation. A summary of the quality flags is provided in Table S1.

### 4.5. Spatial Distribution of Postseismic Deformation

Although postseismic deformation generally decreases with increasing epicentral distance, the relationship is not monotonic. Substantial differences in deformation amplitude occur among stations at comparable epicentral distances, reflecting the heterogeneous and directionally variable deformation field associated with the rupture geometry. Consistent with the predominantly dip-slip faulting mechanism, seven of the nine nonlinear solutions passing the predefined filters occurred in the north component. Only two occurred in the east component, indicating that the resolved postseismic deformation was expressed predominantly in the north–south component.

**Figure 8:**
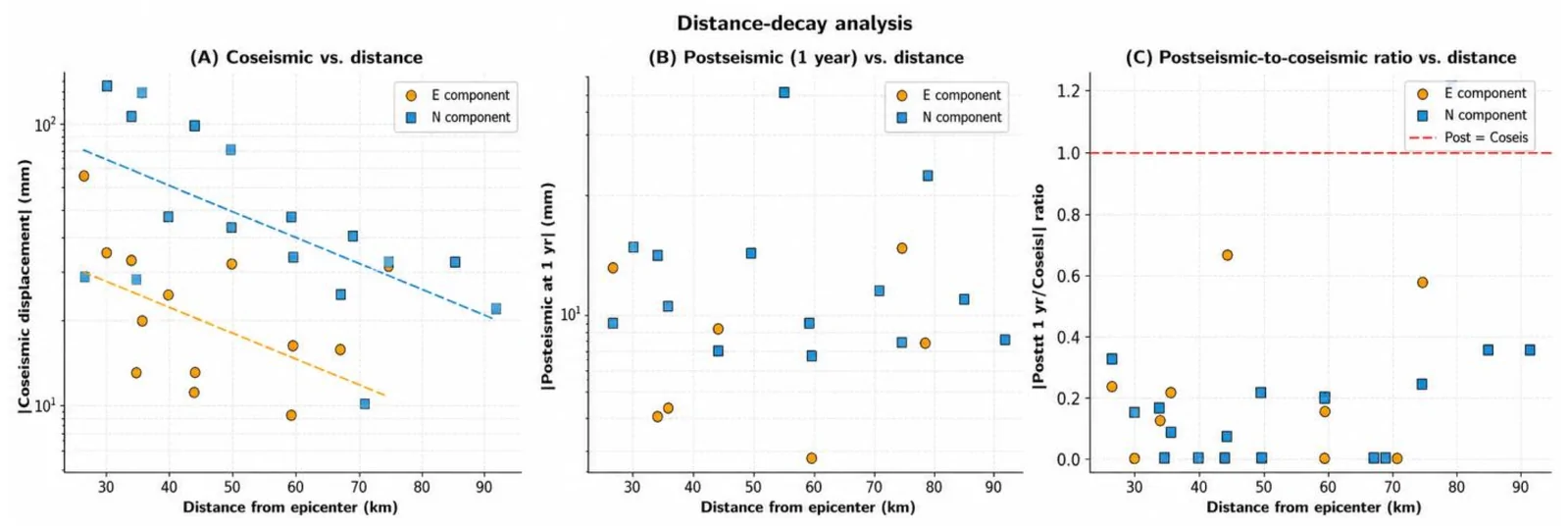
Distance-decay relationships for coseismic displacement, postseismic displacement at 1 year, and the postseismic-to-coseismic ratio as a function of epicentral distance.

Seventeen of the 18 stations (94.4%) are located north of the epicenter. DIDI, the only station south of the rupture, provides a limited constraint on deformation-field asymmetry, but the lack of additional southern stations precludes a comprehensive comparison between hanging-wall and footwall domains. At DIDI-N, the preferred LOGEXP model (A_log = −25.0 mm, τ_log = 109.6 days; A_exp = +43.1 mm, τ_exp = 438.3 days) differs from those selected for the northern stations and requires two distinct temporal components to represent the observations. Because this inference is based on a single southern station, its broader geophysical significance cannot be established from the present dataset alone. The relationship between postseismic displacement amplitude and epicentral distance is presented in Figure 8.

## 5. Discussion

Previous investigations of the 30 October 2020 Mw 7.0 Samos earthquake have focused predominantly on coseismic deformation, fault-source characterization, and rupture modeling [24,26,79,80], whereas its long-term postseismic evolution has remained largely unexplored. By modeling approximately 4.5 years of coordinate time series from a combined network of 18 campaign-style and cGNSS stations surrounding the rupture area, this study provides the regional geodetic characterization of the long-term spatiotemporal evolution of postseismic deformation following the Samos earthquake. The results reveal pronounced spatial variability in postseismic deformation, a broad spectrum of relaxation times, and important constraints imposed by sparse campaign sampling, providing new insights into both the evolution of the Samos postseismic deformation field and the capabilities of campaign GNSS observations for long-term postseismic modeling.

Among the seven single-process LOG and EXP solutions passing the Monte Carlo and goodness-of-fit filters, characteristic relaxation times ranged from 182.6 to 730.5 days, with a median of 438.3 days. The shorter timescales at KBR2-N and SFRH-N may be compatible with relatively rapid aseismic slip during the early postseismic stage and are consistent with the shallow afterslip model proposed by [24]. Three solutions reached the upper campaign-grid boundary of 730.5 days, indicating that the available campaign observations do not resolve timescales beyond the tested range. Long-lasting deformation may include contributions from afterslip and viscoelastic relaxation, which can persist over multi-year to decadal timescales [19]; however, the present data do not permit these mechanisms to be distinguished uniquely. The LOG, EXP, and LOGEXP functions used here are empirical representations of temporal behavior and are not mechanism-specific. At DIDI-N, the preferred LOGEXP model contains a short logarithmic timescale (τ_log approximately 109.6 days) and a longer exponential timescale (τ_exp approximately 438.3 days), indicating that two temporal components are required to represent the time series. Although this behavior may be compatible with more than one postseismic process, parameter trade-offs and the absence of a physics-based deformation model preclude a unique mechanistic interpretation.

LOG decay is typically associated with afterslip, whereas EXP decay is often indicative of viscoelastic relaxation [81]. However, within the present dataset, this expected distinction is not expressed as a simple spatial pattern. The LOG behavior is observed, among the well-constrained solutions, at ZEYT-N, a relatively near-field station located north of the epicenter, and may therefore be interpreted as being compatible with an afterslip-dominated postseismic response. In contrast, stations at comparable near-field distances, such as SIGA-N and SFRH-N, are better represented by EXP models, despite their proximity to the rupture. Thus, the present observations do not show a systematic near-field/far-field separation in which localized afterslip-like behavior dominates the near field and longer-lived relaxation dominates at greater distances. Instead, the preferred temporal behaviors exhibit substantial spatial heterogeneity. This apparent heterogeneity may partly reflect the limited temporal resolution of the campaign GNSS observations. In particular, rapidly decaying afterslip during the early postseismic stage may have occurred before the campaign observations could adequately resolve it, as documented for other earthquakes and consistent with the early postseismic behavior reported for the Samos earthquake [24,62]. The spatial interpretation is further constrained by the offshore setting of the Samos earthquake, which limits the ability to establish a GNSS network with uniform coverage on both sides of the rupture. Consequently, the observed EXP behavior at near-field stations should not be taken as evidence against an early afterslip contribution, nor should the LOG behavior be regarded as uniquely diagnostic of afterslip. Rather, the spatially variable model preferences likely reflect the combined effects of heterogeneous postseismic deformation processes and the temporal and geographical limitations of the available GNSS observations.

Ref. [26] reported that the aftershock distribution following the Samos earthquake was entirely confined to regions of positive coseismic Coulomb stress change, leading them to conclude that short-term postseismic stress transfer played only a negligible role in controlling the spatial distribution of aftershocks. At first glance, this interpretation may appear inconsistent with the postseismic deformation identified in the present study. However, the two observations are not mutually exclusive. For postseismic stress transfer to substantially reorganize regional aftershock activity, afterslip must extend over a sufficiently broad portion of the fault plane. In contrast, afterslip confined to the shallow fault interface or localized near the upper edge of the coseismic rupture may generate measurable surface deformation while producing only limited changes in the regional aftershock distribution. Consistent with this interpretation, Ref. [24] attributed the observed postseismic uplift pattern to shallow afterslip localized near the upper edge of the rupture and suggested that this process may have contributed to the aftershocks clustered along the northern coast of Samos during the first 24 h after the mainshock. Likewise, the postseismic signals resolved from our campaign-style GNSS network are concentrated primarily at near-field stations, providing independent geodetic evidence that is consistent with a spatially localized afterslip process. Another potentially relevant contribution to the observed postseismic deformation is poroelastic rebound. Although poroelastic rebound can contribute appreciably to postseismic deformation in some normal-faulting earthquakes, it is relatively rare for poroelastic rebound to dominate early postseismic deformation [81]. Moreover, recent numerical modelling indicates that poroelastic responses may persist for months to several years, particularly where significant pre-existing fluid overpressure and permeability changes are involved [82]. The offshore setting and extensional tectonic regime of the Samos earthquake may therefore permit post-earthquake fluid migration and localized pore-pressure effects, as demonstrated for other extensional Greek earthquakes [83]. However, no independent geophysical or hydrological observations currently available for the Samos event provide sufficient constraints on crustal fluid overpressure, permeability evolution, or fluid drainage to isolate a poroelastic contribution from the GNSS time series. We therefore do not explicitly model poroelastic rebound but regard it as a potentially unresolved contribution to the observed postseismic deformation.

The spatial distribution of postseismic deformation shows a pronounced concentration of well-resolved nonlinear behavior in the north component: seven of the nine solutions passing the predefined filters occurred in the north component. This pattern is consistent with the kinematics of an E-W-striking normal-fault rupture, for which dip-slip motion is expected to generate predominantly north–south surface displacements. This interpretation is supported by the coseismic displacement vectors reported by [24,26,62]. Only two filtered nonlinear solutions (KBR2-E and IZMI-E) occurred in the east component, indicating a weaker resolved transient signal in the east–west component.

The principal limitation of the present GNSS network is the lack of site coverage south of the rupture. Of the 18 stations included in this study, only one station (DIDI) is located on the southern (footwall) side of the epicenter, whereas the remaining 17 stations are situated north of the rupture on the hanging-wall block. Furthermore, because the Samos earthquake occurred on an offshore fault system, establishing a GNSS network capable of uniformly resolving deformation on both sides of the rupture is inherently challenging. The extensive marine area separating the hanging-wall and footwall blocks severely restricts station distribution, resulting in a substantially less well-constrained network geometry than is typically achievable for continental earthquakes. Consistent with this geometric/topographic limitation, Ref. [24] reported that the coseismic displacement observed at station DIDI (~5 mm southward) was considerably smaller and substantially more uncertain than those recorded at the northern stations. Although this asymmetry documents the coseismic deformation field, it also limits a comprehensive comparison of postseismic deformation between the hanging-wall and footwall domains. The preferred LOGEXP model identified at DIDI-N is consistent with more complex postseismic deformation on the footwall side of the rupture; however, the available observations do not allow this interpretation to be distinguished from increased model uncertainty arising from the sparse station geometry.

The well-constrained solutions identified in this study demonstrate that the postseismic deformation field following the 2020 Samos earthquake is characterized by pronounced spatial variability and temporally heterogeneous relaxation behavior. Nevertheless, an important limitation arises from the temporal resolution of the campaign GNSS observations, which directly affects the detectability of short-lived transient deformation signals. Approximately half of the analyzed station-component time series (50%) were best described by the linear model. Rather than indicating the absence of postseismic deformation, this proportion most likely reflects the inability of the approximately six-month campaign sampling interval to statistically resolve transient deformation components with characteristic timescales shorter than the observation interval from the secular trend. This finding further emphasizes the strongly time-dependent nature of postseismic deformation following the Samos earthquake, for which the detectability of transient processes depends critically on observation timing. cGNSS observations reported by [24,62] provide an important reference in this context. Using the continuous SAMO and SAMU stations, these studies identified a rapidly decaying postseismic transient equivalent to approximately 12–33% of the coseismic displacement that developed within the first 10–30 days after the earthquake and decayed with a characteristic timescale of approximately 30 days. Such a rapidly evolving signal is largely suppressed within the temporal sampling window of campaign-style GNSS observations. Consequently, the high proportion of linear-model selections identified in the present study should be interpreted primarily as a consequence of sparse temporal sampling rather than as evidence for the absence of postseismic deformation.

Several methodological limitations should be acknowledged. The discrete relaxation-time grid used for campaign GNSS time series (182.6, 292.2, 438.3, and 730.5 days) limits the resolution of timescales between and beyond the predefined values. The cGNSS time series were evaluated over a broader grid that included shorter timescales, while the LOGEXP model used separate short logarithmic and longer exponential candidate grids. Although continuous optimization of τ would provide greater flexibility, the limited temporal information in campaign time series produces strong parameter trade-offs and can yield physically implausible solutions. Seasonal terms were not estimated directly in the campaign models. Because campaign observations were concentrated mainly in November and April, unresolved seasonal motion may alias into the estimated transient signals rather than being independently separated. Harmonic analysis of the cGNSS stations indicates that this contribution is generally of millimeter scale and may reach approximately 28% of the expected signal in the north component. While this percentage is non-negligible, the direction of aliasing bias can be assessed from the campaign epoch geometry. November observations introduce a slightly negative seasonal contribution coinciding with the expected postseismic signal, whereas April observations introduce an opposing positive contribution. Because these two effects are of comparable magnitude and partially cancel across the observation epochs, the unresolved seasonal signal is not expected to produce a systematic unidirectional bias in either the estimated amplitudes or the characteristic relaxation times. Instead, it is expected to manifest as increased scatter of approximately 1–3 mm in the north component, which may elevate relaxation-time uncertainty for station components with small postseismic amplitudes. A physically based common-mode correction derived from the continuous stations was not applied because spatial interpolation of seasonal parameters across the topographically and geologically heterogeneous study region would introduce additional uncertainty that is difficult to quantify. As emphasized by [65], discrimination among postseismic mechanisms requires dense and continuous geodetic observations. Campaign GNSS networks are therefore better suited to resolving medium- to long-term deformation than rapidly evolving early transients. Within these limitations, this study provides a regional geodetic characterization of medium- to long-term postseismic deformation following the 2020 Samos earthquake and establishes a quantitative reference for future investigations in the eastern Aegean region.

## 6. Conclusions

This study investigated the long-term temporal evolution of postseismic deformation following the 2020 Mw 7.0 Samos earthquake using campaign and continuous GNSS observations spanning approximately 4.5 years. By integrating pre-earthquake velocity constraints with AICc-based model selection and Monte Carlo observation-perturbation analysis, postseismic deformation was characterized at the station-component level despite sparse campaign sampling. The results reveal a spatially heterogeneous deformation field represented by linear, logarithmic, exponential, and combined logarithmic–exponential behavior. The seven single-process LOG and EXP solutions passing the Monte Carlo and goodness-of-fit criteria yielded characteristic timescales of approximately 183–731 days, with a median of approximately 438 days. The two LOGEXP solutions additionally contained a shorter logarithmic timescale of approximately 110 days and exponential timescales of approximately 292–438 days, demonstrating that the postseismic response was not governed by a single regional relaxation timescale.

The observed spatial variability in preferred model types and relaxation timescales suggests that postseismic deformation following the Samos earthquake cannot be adequately described by a single temporal behavior. Instead, the results indicate that different sectors of the deformation field experienced distinct transient responses, consistent with the complex tectonic setting of the Eastern Aegean and Western Anatolia. Although the available geodetic observations do not allow individual physical mechanisms to be uniquely resolved, the identified spatiotemporal variability is compatible with the contribution of multiple postseismic processes acting with different characteristic timescales. More broadly, this study demonstrates that campaign GNSS observations, when analyzed within an appropriate statistical framework, can provide meaningful constraints on the long-term evolution of postseismic deformation at both near-field and intermediate distances from the rupture.

At the same time, the results highlight the inherent limitations of campaign-based postseismic monitoring. Owing to the sparse temporal sampling, rapidly decaying transient signals occurring during the first weeks or months after the earthquake may remain unresolved, particularly when early afterslip constitutes a substantial fraction of the total postseismic deformation. The limitations imposed by network geometry are further amplified in offshore earthquakes, where the surrounding sea severely restricts the establishment of an optimally distributed geodetic network. Collectively, these findings demonstrate that characterization of postseismic deformation is highly sensitive to observation timing and that a significant fraction of the deformation budget may be accommodated by rapidly decaying transient processes during the earliest postseismic stage. These results further emphasize the inherently time-dependent nature of postseismic deformation and underscore the critical role of appropriate temporal sampling in resolving its full spatiotemporal evolution. Accordingly, future postseismic monitoring strategies should combine dense observations immediately following major earthquakes with sustained long-term geodetic measurements to capture both the rapidly evolving early transients and the longer-term relaxation processes.

## Data Availability

The data presented in this study are available on reasonable request from the first author. The data are not publicly available due to privacy and ethical restrictions.

## Supplementary Materials

The following supporting information can be downloaded at https://www.mdpi.com/article/10.3390/s26175609/s1: Table S1: Postseismic modeling results.
